# Adherence and Willingness to Participate in Cancer Screening Programs Among Women Living in Prison: A Cross-Sectional Study in Southern Italy

**DOI:** 10.3390/healthcare13212735

**Published:** 2025-10-29

**Authors:** Giovanna Paduano, Gabriella Di Giuseppe, Gaia D’Antonio, Marco Ilardi, Giuseppe Nese, Maria Pavia

**Affiliations:** 1 Department of Experimental Medicine, University of Campania “Luigi Vanvitelli”, Via Luciano Armanni 5, 80138 Naples, Italy; giovanna.paduano@unicampania.it (G.P.); gabriella.digiuseppe@unicampania.it (G.D.G.); gaia.dantonio@unicampania.it (G.D.); marco.ilardi@studenti.unicampania.it (M.I.); 2 Unità Operativa Complessa Tutela della Salute in Carcere-ASL Caserta, Via Unità Italiana 28, 81100 Caserta, Italy; giuseppe.nese@aslcaserta.it

**Keywords:** cancer screening programs, women living in prison, adherence, willingness, survey, Italy

## Abstract

**Background/Objectives:** Data on cancer screening programs as well as on the willingness to adhere to these programs in women living in prison (WLP) are lacking. This study investigated the adherence and willingness to undergo cancer screening programs among WLP. **Methods:** This cross-sectional study was conducted from October 2023 to March 2024. **Results:** Overall, 159 WLP were eligible for at least one screening test and 56.8%, 57.6%, and 27.6% had undergone a mammography, PAP-test, and fecal occult blood test (FOBT) in screening programs, respectively. Having undergone a PAP-test for screening purposes was significantly more likely for those in the overweight category, who were experiencing their first detention and were involved in working activities in prison. Moreover, 72.5%, 56.7%, and 72.9% expressed their willingness to undergo mammography, PAP-test, and FOBT for screening purposes if offered in prison, respectively. Willingness to undergo a PAP-test for screening in prison was significantly higher in those in their first experience of detention, who were underweight/healthy weight, who reported correct fruit, vegetable, and protein consumption, and who had expressed willingness to receive vaccinations in prison if offered. Furthermore, older WLP and those with a length of detention of 2–5 years were significantly less willing to undergo a PAP-test. **Conclusions:** The findings of this study have demonstrated that adherence to recommended cancer screening tests is definitely poor in WLP, but have ascertained a strong willingness to participate in cancer screening programs if offered in prison. This is an opportunity that cannot be missed, suggesting that it is imperative to recommend policies aimed at the elimination of barriers for the provision of cancer screening tests as standard preventive care for WLP.

## 1. Introduction

Cancer is the second leading cause of death among European residents, accounting for 22.3% of all deaths, after cardiovascular diseases, with breast cancer being the most common cause of death among European women aged less than 65 years in 2022 [1]. In Italy, in 2023, 395,000 new diagnoses of cancer have been estimated, with 187,000 occurring in women, and it is expected that in the next two decades the absolute annual number of new cancer diagnoses in women will increase, on average, by 0.6% per year [2].

Although data on cancer incidence, prevalence, and mortality in people living in prison (PLP) are scarce, there seem to be evidence that both PLP and those formerly incarcerated have higher cancer mortality than the general population, and a higher incidence of certain cancers, such as cervical, lung, colorectal, and hepatocellular carcinoma, for which effective prevention and screening interventions exist [3,4]. Specifically, there is evidence that cancer mortality is 1.6 times higher in formerly incarcerated men and 1.4 times higher in women compared to the general population [5].

In Europe, one in twenty PLP is a woman [6], representing a minority within the total population of PLP; therefore, since the majority of international research has been mainly focused on PLP as a whole, women living in prison (WLP) represent a distinct understudied population with several unique challenges and needs, which are not specifically addressed by most studies. Specifically, WLP, compared with the general population, experience higher rates of breast (BC) and cervical cancer (CC), likely related to underscreening before incarceration and while in prison [7,8]. A systematic review of WLP reported cervical cancer prevalence at least 100 times higher than in national screening programs, with cervical intraepithelial neoplasia prevalence ranging from 1.13 to 5.46 compared to the community [9]. Furthermore, studies documented carcinoma in situ of the cervix as the most common diagnosis among incarcerated women, representing 83% of cancer cases [7].

Moreover, a recent report by the WHO has provided evidence of cancer and cardiovascular health inequities in prison settings, suggesting that improvement in cancer management in prison through preventive health services, such as prison health screening programs, can promote health and reduce costs for health systems [10].

Health disparities in this population are also recognized in Italy, where, by legislation, health services provided to PLP should be the same as those delivered to the general population [11]. Specifically, the Legislative Decree 230/1999 establishes that PLP have the right to healthcare standard equivalent to free citizens, with services covered by regional health authorities, funded by the National Health Service (NHS), which collaborate with prison administrations to ensure equitable healthcare provision [12]. In particular, cancer screening programs are included in the Essential Levels of Care [13], with BC, CC, and colorectal cancer (CRC) screenings being freely and proactively offered to all age-eligible individuals.

For PLP, detention is often the first opportunity, or even the only chance, to access preventive health services [14,15], but comprehensive and systematic data on the provision of cancer screening programs as well as on the willingness to adhere to these programs in WLP are scarce, with most information pertaining to adherence to CC screening, and showing percentages ranging from 46.4% to 83% [16,17,18,19].

This study, as part of a larger project investigating the needs and the delivery of prevention programs among PLP in Southern Italy, aims to evaluate (1) adhesion to cancer screening programs in WLP; (2) whether cancer screening programs are provided in prison; (3) the willingness of WLP to participate to cancer screening programs if they are organized in prison.

## 2. Materials and Methods

### 2.1. Study Design 

The present cross-sectional study was conducted from October 2023 to March 2024 among all WLP aged 25–69 years old who were eligible for at least one of the national cancer screening programs from two prisons in the geographical area of the Campania region, Southern Italy. This investigation was part of a larger project developed by the University of Campania, “Luigi Vanvitelli” (Naples, Italy), and the Joint Operational Unit for “Health Protection at Prison Institutions” (Caserta, Italy), focused on the analysis of several health-related issues in the detained population [16,20,21,22]. WLP in the Campania region are hosted in five facilities and are allocated within minimum-security-level sections. One of the facilities is completely dedicated to WLP, hosting almost half of the WLP in the entire region, and in the other four, WLP are hosted in specific women-dedicated sections. In these four facilities, the number of WLP is similar, ranging from 37 to 55. According to the sampling plan, the facility entirely dedicated to WLP, and one randomly chosen WLP section of the remaining four facilities were selected for this study. All WLP fulfilling the inclusion criteria were recruited in this study.

### 2.2. Data Collection

Before starting the survey, an invitation letter was sent to prison directors to arrange an informational meeting. During this meeting, they received a letter outlining the study protocol, the project objectives, and the data collection methods to obtain their consent to conduct the survey. Once the approval was obtained, a team of trained researchers approached potential participants to explain the purpose of this study, indicating that the questionnaire completion was voluntary and strictly confidential, and asking for their participation. Before the beginning of the interview, each WLP provided verbal informed consent, which was considered more appropriate in this population due to potential literacy, language, and comprehension barriers, as well as to avoid fear of signing documents, which can reduce trust and consequent participation in surveys, as highlighted in previous studies [23,24,25]. To ensure anonymity, each participant was assigned an identification code, and only one officer had the list of codes associated with the participant’s name.

WLP housed in the special psychiatry unit (8 subjects), who were unable to give informed consent due to substantial cognitive impairment, were excluded from the survey. This exclusion was necessary for both ethical and safety reasons, as prison directors did not allow access to this unit due to security protocols and institutional policies. Following the participants’ consent, a trained researcher conducted face-to-face interviews on different days and at various times throughout the week. The interview with each participant was performed in a semi-private area at the jail, with the interviewer and participant seated on opposite sides of a table. A correctional officer remained at a distance of approximately 10 m during the interviews to ensure security and privacy. The interview and the compilation of the questionnaire by the research investigators lasted approximately 20 min. 

### 2.3. Survey Instrument

The interview was based on a questionnaire developed according to a comprehensive literature review focusing on prison environments, and the items of the questionnaire were validated in previous investigations conducted by some of us regarding adherence and willingness to participate in organized cancer screening programs in the general population [26,27] and in the prison setting [16,20,21].

The questionnaire was composed of four sections, introduced by a preface outlining the survey’s objectives and the measures taken to ensure that participants could not be identified from the collected information. The first was on socio-demographic, detention-related, and anamnestic characteristics of WLP (age, nationality, sexual orientation, marital status, sons/daughters, education level, occupation before and during detention, length of detention, living arrangements [individual or shared cells], history of chronic diseases, and weight and height to obtain Body Mass Index [BMI]).

The second explored lifestyle behaviors including smoking status, number of cigarettes, alcohol consumption, drug abuse, physical activity status, daily consumption of fruit and vegetables, daily portions of dietary protein sources, and frequency of snack and sweet consumption. Dietary variables were included because, despite the provision of standardized institutional meals, the WLP in the sample had access to self-cooking facilities and food provided by authorized family visits, leading to variation in dietary patterns. 

The third section investigated participation in the organized cancer screening programs, focusing on mammography for BC screening, the Papanicolaou test (PAP-test) and Human Papillomavirus DNA-test (HPV DNA-test) for CC screening, and the fecal occult blood test (FOBT) for CRC screening. Screening tests in prison were organized in coordination with regional health authorities, with PAP-test and FOBT generally conducted within prison facilities. For BC screening, mammography was performed directly in prison using a portable unit or through scheduled sessions in authorized regional public centers. Emphasis was placed on the role of health services dedicated to WLP in facilitating access to these screening programs, as well as on their willingness to participate and the reasons influencing their decisions. In the last section, willingness to uptake recommended vaccination (Hepatitis B, Diphtheria/tetanus/pertussis, Meningococcus ACW135Y, Meningococcus B, Measles/mumps/rubella/varicella, Hepatitis A, Pneumococcal, Influenza, and Herpes Zoster) in the eligible subjects, if offered in prison, was also investigated.

In all sections, information was collected using closed-ended questions and a multiple-choice answer format. Furthermore, physical activity status was assessed with the International Physical Activity Questionnaire—Short Form (IPAQ-SF) [28,29]. The IPAQ-SF, with seven items, allowed for the calculation of Metabolic Equivalent of Task (MET) values by measuring the frequency, duration, and intensity of physical activities over the previous seven days. The total energy expenditure was expressed as MET-minutes per week (MET-min/week), categorized into three activity levels: inactive (<700 MET-min/week), sufficiently active (700–2519 MET-min/week), and active/very active (>2520 MET-min/week). 

Alcohol-related disorders were investigated using the Alcohol Use Disorders Identification Test-Concise (AUDIT-C), a validated questionnaire that consists of three items: “How often do you consume alcoholic drinks?”; “On days when you drink, how many alcoholic drinks do you consume on average?”; “How often do you drink six or more glasses of alcohol on a single occasion?” [30]. Each AUDIT-C question had 5 answer choices valued from 0 to 4 points, and the final score was from 0 to 12. The test reveals an average risk of developing an alcohol-related disorder (consumer not at risk, harmful consumption, or alcohol dependence).

### 2.4. Statistical Analysis

First, descriptive statistics, including frequencies, means, and standard deviations, were used to summarize retrieved information. Then, two stepwise multivariate logistic regression models were designed for the following outcomes: PAP-test uptake in a screening program (Model 1), which was dichotomously recorded as 1 if the answer was “yes” and 0 if it was “no/do not remember,”; and willingness to undergo PAP-test for screening purposes if offered in prison (Model 2), which was dichotomized as 1 if the answer was “yes” and 0 if it was “no/not sure”. The models were developed according to Hosmer and Lemeshow’s strategy [31] that includes the following steps: (1) bivariate analysis of each variable considered, using the appropriate test statistic (chi-square test, Fisher’s exact test, and Student’s *t*-test) with the normality of continuous variables being tested by the Shapiro–Wilk test; (2) choice of the way to include independent variables in the model (continuous, ordinal, or categorical) by taking into account how each of these ways better fitted the data at the bivariate analysis; (3) inclusion in the model of any variable whose bivariate test had a value of *p* ≤ 0.25 or that was judged to potentially have an influence on the investigated outcomes; (4) the values for variables’ entry and removal in the final models were, respectively, *p* = 0.2 and *p* = 0.4 throughout the stepwise selection procedure.

The following independent variables were selected for both models: institution (Prison 1 = 0; Prison 2 = 1), age in years (continuous), marital status (unmarried/widowed/separated/divorced = 0; married/cohabitant = 1), sons/daughters (no = 0; yes = 1), education level (ordinal) (none/primary school = 0; middle school = 1; high school/university degree = 2), occupation before detention (no = 0; yes = 1), first detention (no = 0; yes = 1), length of detention, years (≤1=1; 2–5 = 2; >5 = 3), working activity in prison (no = 0; yes = 1), at least one chronic disease (no = 0; yes = 1), BMI category (underweight/healthy weight = 1; overweight = 2; obese = 3), smoking status (never smoker = 1; former smoker = 2; current smoker = 3), alcohol consumption (never = 1; not being at risk of alcohol abuse = 2; being at risk of alcohol abuse = 3), physical activity status (ordinal) (inactive = 1; minimally active = 2; active/very active = 3), at least five daily portions of fruit and vegetables (no = 0; yes = 1), two daily portions of dietary protein sources (no = 0; yes = 1), rare snack and sweet consumption (no = 0; yes = 1). In the second model, PAP-test uptake (no/do not remember = 0; yes = 1), willingness to receive at least one recommended vaccination in prison (no/not sure = 0; yes = 1), and willingness to participate to at least one intervention about healthy lifestyle in prison (no/not sure = 0; yes = 1) were also added. Adjusted odds ratio (OR) and 95% confidence intervals (CIs) were calculated. All reported *p* values are two-tailed, and a value ≤ 0.05 is considered statistically significant. Multicollinearity was checked through the variance inflation factor (VIF), goodness-of-fit through the Hosmer–Lemeshow test, and discrimination through the area under the ROC curve (AUC) metric. Moreover, sensitivity analysis was performed excluding flagged observations. All analyses were performed using Stata software, version 17 [32].

### 2.5. Pilot Study

Prior to the beginning of the survey, the questionnaire was pre-tested on a random sample of 50 women to assess the clarity, reliability, and feasibility of the questions. Training of interviewers was performed to evaluate inter-rater reliability, the results of which were deemed satisfactory after completion of the pilot study. The pilot study did not reveal any issues requiring modification, and therefore responses from these women were included in the final analysis.

### 2.6. Ethics

This study was approved by the Ethics Committee of the University of Campania “Luigi Vanvitelli” (protocol code: N.0029553/12 October 2023). All data were collected using a pre-coded questionnaire without recording participants’ names. All analyses were performed on aggregate data to ensure complete anonymization. Data were stored on password-protected systems with access restricted to authorized research team members only.

## 3. Results

### 3.1. Socio-Demographic, Detention, Anamnestic, and Lifestyle Characteristics of the Participants

Of the 215 WLP who were present in the selected facilities, 8 were excluded because they were allocated to the special psychiatry unit, and of the remaining 207 who were invited to participate, a total of 172 agreed to be interviewed, with a response rate of 83.1%. Among these, 159 were eligible for at least one screening test (25–69 years). In particular, 95 women were eligible for mammography (45–69 years), 151 for PAP-test (25–64 years), and 58 for FOBT (50–69 years). The main socio-demographic, detention, anamnestic, and lifestyle characteristics of WLP are shown in Table S1 in the Supplementary Materials.

The mean age was 46.8 years (SD ± 10.2), most of the respondents (91.2%) were Italian, only 20.1% had attained a high school or university degree, almost half (49.7%) were married or cohabitant, 82.4% had at least one child, and 62.3% were employed before detention. Almost all (95%) lived in shared cells, 37.7% were involved in some working activity, 69.6% were in their first episode of detention, and the mean time spent in prison was 2.6 years. Overall, 37.1% of the participants reported as being affected by at least one chronic disease, and the most frequent were cardiovascular diseases (20.1%) and diabetes (6.9%). Regarding the BMI category, 24.5% were classed as overweight and 33.1% were classed as obese. Current smokers were the most represented group (69.8%), with a mean number of 20.1 cigarettes smoked a day, 17.9% were considered at risk of alcohol abuse, while 53.2% declared they had never consumed alcohol, and only 27.4% were active or very active. A total of 27.7% participants reported lifetime drug use, with cocaine (61.4%) and marijuana (43.2%) being the most frequently used substances; moreover, intravenous drugs were reported by 16% of drug users. More than two-thirds (69.2%) reported healthy dietary habits with regard to the consumption of at least five daily portions of fruit and vegetables, 41.5% consumed two daily portions of dietary protein sources, and more than half (52.2%) declared rare snack and sweet consumption (Table S1).

### 3.2. BC Screening Behavior and Willingness to Participate in BC Screening Activities

**Figure 1:**
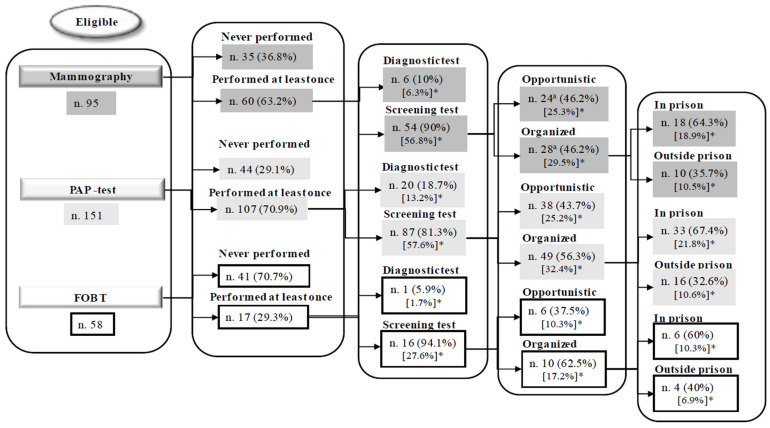
Experience of mammography, PAP-test, and FOBT in detained women. * Percentages in square brackets refer to the total number of eligible women. ^a^ Number of each item may not add up to total number of study population due to missing values.

Overall, of the 95 eligible WLP, 60 (63.2%) had previously undergone a mammography, 6.3% as a diagnostic exam and 56.8% (95% CI = 46.3–67%) for screening purposes; for those who had undergone mammography for screening, 25.3% participated in an opportunistic procedure, and 29.5% in an organized program—18.9% in prison and 10.5% outside of prison (Figure 1). The adherence to the screening interval of two years was reported by 35 (36.8%) of the eligible WLP. Main reported reasons for not having undergone mammography were not having been advised (37.1%), perceived absence of health problems (20%), and lack of time (11.4%). When WLP were asked whether they had been invited to participate in a BC screening program in prison, 26 (27.4%) reported as having been invited and, of these, 80.8% had undergone mammography, whereas when those who had not been invited were asked whether they would participate in BC screening programs if offered in prison, 72.5% expressed their willingness to undergo mammography for screening purposes. Among those who were not willing to participate, the more frequently reported reasons were perceived absence of health problems (40%) or considering the test to be useless (20%). Participation and willingness to participate in BC screening among WLP according to several characteristics are reported in Table S1. None of the tested characteristics significantly predicted participation in BC screening or willingness to participate, whereas significant differences in previous participation were revealed across the investigated prisons (*p* = 0.001).

### 3.3. CC Screening Behavior and Willingness to Participate in CC Screening Activities

Overall, 151 WLP were eligible, and 107 (70.9%) had previously undergone a PAP-test, 13.2% for diagnostics and 57.6% (95% CI = 49.3–65.6%) for screening purposes; for those who had undergone a PAP-test for screening, 25.2% had participated in an opportunistic test, while 32.4% in an organized program, 21.8% in prison and 10.6% outside of prison (Figure 1). The adherence to the screening interval of three years was reported by 60 (39.7%) of the eligible WLP. Main reasons for not having undergone a PAP-test were perceived absence of health problems (37.5%) and not having been advised (30%). Overall, 47 (31.1%) reported as having been invited to participate in a CC screening program in prison, and 87.2% of them had accepted to undergo a PAP-test; moreover, among those who had not been invited, 56.7% expressed their willingness to undergo a PAP-test for screening purposes if offered in prison.

For those who were not willing to participate, the more frequently reported reasons were absence of health problems, being afraid of discovering the disease, or considering the test to be useless (20%). Participation and willingness to participate in CC screening according to the main characteristics are reported in Table S1. Having undergone a PAP-test for screening purposes was significantly more likely among more educated women (*p* = 0.037), experiencing their first detention (*p* = 0.010), and for those in the overweight category (*p* = 0.041), whereas willingness to undergo a PAP-test in prison was significantly more likely in those who reported consumption of at least five daily portions of fruit and vegetables (*p* = 0.016).

Among the 145 eligible WLP, only 8 (5.5%) had ever undergone an HPV-DNA test, 2 (1.4%) for diagnostics and 6 (4.1%) for screening purposes. Among those who had performed an HPV-DNA test for screening, two (1.4%) participated in an opportunistic test, while four (2.8%) in an organized program, two (1.4%) in prison and two (1.4%) outside of prison. The adherence to the screening interval of five years was reported by all of the WLP. The main reasons for not having undergone an HPV DNA-test were perceived absence of health problems (53.8%) and not having been advised (36.8%).

### 3.4. CRC Screening Behavior and Willingness to Participate in CRC Screening Activities

Overall, out of 58 eligible WLP, 17 (29.3%) had previously undergone a FOBT, 1.7% for diagnosis and 27.6% (95% CI = 16.7–40.9%) for screening purposes; of these, 10.3% participated in an opportunistic procedure, and 17.2% in an organized program, 10.3% in prison and 6.9% outside of prison (Figure 1). The adherence to the screening interval of two years was reported by 10 (17.2%) of the eligible WLP. The main reported reasons for not having undergone FOBT were perceived absence of health problems (61.5%) and not having been advised (25.6%). Invitation to participate in the CRC screening program in prison was reported by 10 (17.2%) of the eligible women, and 80% reported as having accepted to undergo, while 72.9% expressed their willingness to undergo FOBT for screening purposes if offered in prison. All of those who were not willing to participate reported that the reason was they did not feel at risk.

Participation and willingness to participate in CRC screening according to several characteristics are reported in Table S1. Having undergone FOBT for screening purposes was significantly more likely in those at their first detention experience (*p* = 0.027), who consumed at least five daily portions of fruit and vegetables (*p* = 0.045), and who were in the overweight category (*p* = 0.044). Willingness to uptake FOBT in prison was significantly more likely in those who did not have an occupation before detention (*p* = 0.032).

### 3.5. Multivariate Analysis

The results of the multivariate logistic regression models are shown in Table 1. Having undergone a PAP-test for screening purposes was four times more likely in women in the overweight category compared to women in the obese category (OR = 4.08, 95% CI = 1.48–11.25), 2.7 times in WLP experiencing their first detention compared to those who had multiple prison experiences (OR = 2.67, 95% CI = 1.12–6.37), and 2.4 times more likely in those involved in working activities in prison (OR = 2.45, 95% CI = 1.04–5.76) (Model 1 in Table 1).

The odds of willing to undergo a PAP-test in WLP detained for the first time were more than six times those of WLP who had multiple prison experiences (OR = 6.57, 95% CI = 1.26–34.22); in underweight/healthy-weight women, they were more than five times those of WLP in the obese category (OR = 5.13, 95% CI = 1.14–23.21); in those who reported correct fruit and vegetables, they were more than six times (OR = 6.47, 95% CI = 1.31–32.12); in those who had correct protein consumption, they were thirteen times higher (OR = 13.09, 95% CI = 1.99–86.05); and those who expressed willingness to receive vaccination if offered in prison were more than one-hundred times higher than those of WLP who were not willing to receive vaccination in prison (OR = 119.07, 95% CI = 11.34–1250.19). Moreover, these odds were 12% (OR = 0.88, 95% CI = 0.81–0.98) lower in older compared to younger women, and 86% lower in those with a length of detention of 2–5 years compared to those with more than 5 years detention in prison (OR = 0.14, 95% CI = 0.02–0.82). Finally, significant differences in willingness were also detected in the different institutions (Model 2 in Table 1).

In both models, no relevant collinearity was found, with mean and maximum VIF being 1.55 and 3.12 in Model 1, and 1.94 and 4.46 in Model 2 (Table 1). Moreover, good calibration was revealed by the Hosmer–Lemeshow goodness-of-fit test for both Model 1 (χ^2^ (8) = 7.19, *p* = 0.516) and Model 2 (χ^2^ (8) = 2.24, *p* = 0.973), as well as satisfactory discrimination, with the AUC being 0.710 (95% CI = 0.624–0.797) in Model 1 and 0.884 (95% CI = 0.817–0.951) in Model 2 (Table 1). Finally, sensitivity analysis showed that the results were not substantially different after the exclusion of flagged cases; therefore, it may be concluded that the initial findings were robust.

## 4. Discussion

The results of this study have documented that the provision of cancer screening to WLP is far from being satisfactory, despite the fact that detention in prison may represent an opportunity for health promotion and cancer prevention in this hard-to-reach population. For all the investigated screening tests, the ever-attending prevalence was poor and, as expected, it was higher for the PAP-test (57.6%), followed by mammography (56.8%), and far lower for FOBT (27.6%). Indeed, the results of the National Observatory on screening in the general population of Southern Italy [2] confirm the low rate of adherence to CRC screening compared to BC and CC screening, and data on cancer screening in the general population of women in the same area show higher rates for CC and BC screening, 85.4% and 78.2%, respectively [26], whereas adherence to FOBT was analogously scarce (25%) [27].

The comparisons with studies performed on incarcerated women show that, for mammography, the adherence in this study was higher compared to the American study by Pickett et al. (42.1%) [8], but lower than the study by Binswanger et al. (66%) [17], whereas for PAP-test in prison, studies have reported that ever-attendance ranged from 46.4% to 83% [16,17,18,19], positioning the findings of this study in the lower bound; moreover, for FOBT, the results are in line with the only previous study in the same population (25%) [17].

These findings are even more concerning when considering the adherence to the recommended time interval since the last screening test; given that it ranged from 39.7% for the PAP-test to 17.2% for FOBT, even in this case, it is significantly lower than the adherence measured in women of the general population in the same area [26,27], and for WLP. Indeed, the literature exploring the same population reports mammography in the previous 2 years to range from 34.1% [33] to 58% [19]; PAP-test in the previous 3 years from 62% [34] to 90% [17]; and FOBT in the previous two years was from 22.9% [33] to 31% [17].

The discrepancies observed in adherence rates, particularly compared to studies from North America, reflect structural differences in the organization of prison health care. In Italy, prison health services are integrated into the public NHS, in line with the European principle of equivalence of care and equity, which aims to guarantee the same access to preventive services as in the community [35]. These organizational differences could explain the variability across international studies and highlight the role of health policies and detention conditions in shaping participation in screening.

Despite these concerning findings, there are also two very promising results to underscore in this study: first of all, the great majority of women who were offered cancer screening tests in prison reported to have undergone them, namely more than 80%; and, second, the willingness to undergo screening tests was high, ranging from 56.7% for the PAP-test to 72.9% for FOBT among women who were not offered it in prison, in line with similar studies conducted in PLP [17,19]. These findings reveal the potential extraordinary role that health services dedicated to PLP may have to reduce inequalities in cancer prevention activities; this is confirmed by another very interesting result of this study, showing that for all investigated screening tests, the attendance within prison, although low compared to other similar studies—which report PAP-test adherence ranging from 46.4% to 83% [16,17,18,19]—it is always higher than that reported outside of prison in organized screening programs. However, the results investigating the provision of cancer screening tests within prisons counterbalanced those on participation and willingness to participate, since they have shown that for all screening tests, invitation has been reported to be very low, ranging from 17.2% for FOBT to 31.1% for PAP-test, far below the rate of invitations provided to the general population within organized programs in Southern Italy [2]. This is alarming since PLP are under the responsibility of the prison authority for the provision of health services, and there is evidence that cancer diagnoses are, on average, performed at a later stage in PLP compared to the general population [36]. The low invitation rates are concerning and have been reported to be related to structural barriers typical of the correctional health system, including limited budgets dedicated to health prevention activities, staffing constraints, and transport availability [4,37,38].

The analysis of reported reasons for not having undergone screening tests may help in the identification of barriers and facilitators to adherence to screening programs and may assist in the development of strategies to increase uptake. For all investigated tests, the most frequently reported reasons for not having undergone tests and for not being willing to undergo if offered in prison were not having been advised by a physician and not considering themselves to have a health problem. Indeed, in line with these results, a recent review of the literature investigating factors influencing the uptake of BC, CC, and CRC tests in the general population has revealed that the greatest collective facilitator to uptake was recommendation by a healthcare provider—without significant differences among the specific screening tests—whereas among the most encountered barrier was a general lack of knowledge surrounding cancer screening programs [39]. In the same publication, a key element for the success of interventions in this field was “building trusted relationships” [39], and this achievement is feasible for WLP given the opportunity to reach them while they are in custody.

Findings of the multivariate logistic regression analysis indicated that detention-related characteristics, such as first experience of detention and working activities in prison, were recognized as significant determinants of having undergone a PAP-test. Specifically, it has been shown that those who were in their first experience of detention and who were involved in working activities in prison were significantly more likely to have undergone a PAP-test. It is interesting that working activity has been found to be a predictor of good self-rated health status and of self-confidence in the ability to protect from COVID-19 infection in detained subjects in two studies performed by some of us in the same area [21,22], confirming that working activity may produce a sense of well-being in PLP, promoting higher attention also to preventive activities such as the PAP-test. Moreover, PLP at their first incarceration were also more likely to be satisfied by healthcare provided in prison in the same study [22] and the Authors suggested that since incarceration provides easier access to health services for people who often face substantial barriers to accessing healthcare in the community, those in their first incarceration may perceive healthcare services provided in prison as more satisfactory compared to those in the community [22]. Indeed, we found that in this population, provision of the PAP-test was more frequent inside prison than outside.

Several protective or preventive activities, being proxies of a healthy lifestyle, such as correct fruit, vegetable, and protein consumption, willingness to undergo recommended vaccinations if offered in prison, as well as not being in the obese weight category, were demonstrated as determinants of willingness to undergo a PAP-test. This is a promising result, underlining that the promotion of a healthy lifestyle in prison would have an impact on a broader pattern of preventive and protective activities. Moreover, the findings that those with a longer duration of detention were more willing to undergo a PAP-test may be indirect evidence of the role that health services provided to WLP may have in the education of preventive activities.

### Limitations

Interpretation of the findings of this survey should consider the role of some potential limitations. First, the choice of the cross-sectional design precludes the evaluation of the cause–effect relationship between determinants and outcomes; moreover, the failure to define the temporal relationship between independent and dependent variables by design does not allow us to exclude the potential for reverse causality. Second, no objective sources for the assessment of cancer screening attendance were used, since adherence was self-reported, and it is well-known that subjective reporting may be affected by social desirability or recall bias, as well as lack of self-awareness, leading to over- or underestimation of results. Although this kind of bias cannot be excluded, mitigation of social desirability has been introduced by anonymous reporting, whereas recall and lack of self-awareness bias appear to be unlikely, since cancer screening activities are highly significant events, which are quite difficult to forget. Third, no information was available on those who refused to participate; therefore, selection bias cannot be excluded, although the large participation shown in the high response rate has substantially reduced the risk of relevant distortions of the estimates, since it is plausible to argue that the results of the survey would not have been substantially changed by the inclusion of the non-respondents. Fourth, WLP in this study were enrolled from only two prisons in Southern Italy, and it is conceivable that there is potential for selection bias. However, they represent most of the WLP detained in the Campania region; therefore, we believe they are highly representative of the southern context, although the generalizability of the results to the wider population of WLP in Italy and in Europe should be made with caution, and future studies should consider multi-site sampling across different regions to enhance external validity. Fifth, we did not investigate prison administration views on screening, which could help to understand discrepancies in the provision of appropriate preventive health care. It would be interesting to dedicate future research to exploring this perspective. Sixth, investigation of determinants was feasible only for the adherence and willingness to undergo CC screening due to the limited number of eligible women to BC and CRC screening. Moreover, the small sample size has been responsible for the extreme variability in the estimates, as suggested by the wide confidence intervals of the odds ratios in the multivariate models, and this lack of power may have hidden further associations between determinants and the outcomes of interest. Analogously, the limited sample size did not allow a full exploration of potential interaction effects among all the variables, which should be addressed in future studies with larger cohorts. Despite these limitations, this study has made available valuable insights into the critical prevention needs of a vulnerable and understudied population, which would be helpful for the development of tailored prevention programs.

## 5. Conclusions

In conclusion, the findings of this study have demonstrated that, even with some screening-specific differences, adherence to recommended cancer screening tests is definitively poor in the investigated women, coupled with a scarce provision of these programs in the explored context. The results have also ascertained a strong willingness to participate in cancer screening programs, as well as large participation when they are offered in prison. This positive attitude of WLP is an opportunity that cannot be missed, suggesting that it is imperative to recommend policies aimed at the elimination of barriers for the provision of cancer screening tests—such as the training of the prison staff not only for the treatment of diseases but also on preventive interventions—as well as the systematic and regular organization of screening campaigns to promote cancer screening programs as standard preventive care for WLP.

## Figures and Tables

**Table 1:** Multiple logistic regression analysis investigating PAP-test uptake in a screening program (Model 1) and willingness to undergo PAP-test in prison (Model 2) according to several characteristics.

° Model 1. PAP-Test Uptake in a Screening Program			
Log likelihood = −82.2, χ^2^ = 23.1 (7 df), *p* = 0.002, Pseudo R^2^ = 0.123, mean VIF = 1.55, max VIF = 3.12, Hosmer–Lemeshow χ^2^ (8) = 7.19, *p* = 0.516, AUC = 0.710 (95% CI = 0.624–0.797), No. of obs = 139			
**Variable**	**OR**	**95% CI**	***p***
**Body Mass Index (BMI) category**			
Obese	1.00 *		
Overweight	4.08	1.48–11.25	0.007
Underweight/healthy weight	Backward elimination		
**First detention**			
No	1.00 *		
Yes	2.67	1.12–6.37	0.037
**Working activity in prison**			
No	1.00 *		
Yes	2.45	1.04–5.76	0.041
**Education level (ordinal)**	1.61	0.91–2.84	0.102
**Occupation before detention**			
No	1.00 *		
Yes	0.49	0.21–1.13	0.093
**Length of detention, years**			
>5	1.00 *		
2–5	Backward elimination		
≤1	1.47	0.62–3.45	0.381
**At least 5 daily portions of fruit and vegetables**			
No	1.00 *		
Yes	1.49	0.65–3.43	0.352
**^+^ Model 2. Willingness to undergo PAP-test in prison**			
Log likelihood = −36.15, χ^2^ = 57.02 (17 df), *p* < 0.00, Pseudo R^2^ = 0.411, mean VIF = 1.94, max VIF = 4.46, Hosmer–Lemeshow χ^2^ (8) = 2.24, *p* = 0.973, AUC = 0.884 (95% CI = 0.817–0.951), No. of obs = 95			
**Variable**	**OR**	**95% CI**	***p***
**Institution**			
Prison 1	1.00 *		
Prison 2	17.42	1.95–155.32	0.010
**Age, years (continuous)**	0.88	0.81–0.98	0.015
**First detention**			
No	1.00 *		
Yes	6.57	1.26–34.22	0.025
**Length of detention, years**			
>5	1.00 *		
2–5	0.14	0.02–0.82	0.029
≤1	Backward elimination		
**BMI category**			
Obese	1.00 *		
Overweight	2.04	0.39–10.73	0.398
Underweight/healthy weight	5.13	1.14–23.21	0.034
**At least 5 daily portions of fruit and vegetables**			
No	1.00 *		
Yes	6.47	1.31–32.12	0.022
**Two daily portions of dietary protein sources**			
No	1.00 *		
Yes	13.09	1.99–86.05	0.007
**Willingness to receive recommended vaccinations in prison**			
No	1.00 *		
Yes	119.07	11.34–1250.19	<0.001
**Marital status**			
Unmarried/widowed/separated/divorced	1.00 *		
Married/cohabitant	0.49	0.12–2.02	0.330
**Sons/daughters**			
No	1.00 *		
Yes	2.89	0.43–19.67	0.276
**Education level (ordinal)**	0.41	0.15–1.11	0.080
**At least one chronic disease**			
No	1.00 *		
Yes	4.68	0.94–23.34	0.278
**Working activity in prison**			
No	1.00 *		
Yes	0.36	0.06–2.27	0.278
**Alcohol consumption (Audit-C)**			
Being at risk of alcohol abuse	1.00 *		
Not being at risk of alcohol abuse	4.52	0.45–45.54	0.200
Never	2.94	0.31–28.24	0.349
**Willingness to participate in health promotion interventions in prison**			
No	1.00 *		
Yes	1.01	0.99–1.01	0.178

* Reference category. ° The following variables were deleted by the stepwise procedure: age in years (continuous), sons/daughters, marital status, at least one chronic disease, smoking status, alcohol consumption, two daily portions of dietary protein sources, rare snack and sweet consumption, physical activity status (ordinal), institution. ^+^ The following variables were deleted by the stepwise procedure: occupation before detention, smoking status, physical activity status, rare snack and sweet consumption, PAP-test uptake.

## Data Availability

The raw data supporting the conclusions of this article will be made available by the authors, without undue reservation.

## Supplementary Materials

The following supporting information can be downloaded at https://www.mdpi.com/article/10.3390/healthcare13212735/s1, Table S1: Socio-demographic, detention, anamnestic, and lifestyle characteristics of detained women participating in this survey and related associations with previous participation and willingness to undergo cancer screening programs.

## Abbreviations

The following abbreviations are used in this manuscript:

BC	Breast cancer
CC	Cervical cancer
CRC	Colorectal cancer
FOBT	Fecal Occult Blood Test
HPV DNA-test	Human Papillomavirus DNA test
PLP	People living in prison
WLP	Women living in prison
