# Supplementary material for: Adherence and Willingness to Participate in Cancer Screening Programs Among Women Living in Prison: A Cross-Sectional Study in Southern Italy
Source: Healthcare (Basel). 2025 Oct 29;13(21):2735. doi: 10.3390/healthcare13212735 (PMC12610190; doi:10.3390/healthcare13212735)
Supplement: Supplementary file 1 [file healthcare-13-02735-s001.zip › Table 1_Supplementary Materials.pdf]

Table S1. Socio-demographic, detention, anamnestic, and lifestyle characteristics of detained women participating in the survey and related association with previous participation and willingness to undergo cancer screening programs.

			**Mammography**		**PAP-test**[c]		**FOBT**[d]	
**Characteristics**	**Total (N:159)**		**Uptake in a screening program (N:95 Eligible)** [#]	**Willingness to undergo in prison (N:65 Eligible)** ▲	**Uptake in a screening program (N:151 Eligible)**•	**Willingness to undergo in prison (N:104 Eligible)** ▲	**Uptake in a screening program (N:58 Eligible)**◊	**Willingness to undergo in prison (N:48 Eligible)**▲
			**Yes** (N:54;56.8%)	**Yes** (N:50;72.5%)	**Yes** (N:87;57.6%)	**Yes** (N:59;56.7%)	**Yes** (N:16;27.6%)	**Yes** (N:35;72.9%)
Socio-demographics	**N**	%	**N (%)**	**N (%)**	**N (%)**	**N (%)**	**N (%)**	**N (%)**
**Age groups, years**								
25-44	64	40.3	-	-	40 (62.5)	21 (55.3)	-	-
45-49	37	23.2	17 (45.9)	19 (70.4)	19 (51.4)	17 (65.4)	-	-
≥50	58	36.5	37 (63.8)	31 (73.8)	28 (56)	21 (52.5)	16 (27.6)	35 (72.9)
			χ^2=2.05, df=1, *p*=0.152	χ^2=0.097, df=1, *p*=0.755	χ^2=0.79, df=2, *p*=0.670	χ^2=1.12, df=2, *p*=0.572	$\chi^2$for trend =2.51, *p*=0.113	-
**Nationality**								
Italians	145	91.2	51 (57.9)	46 (74.2)	79 (57.7)	54 (56.3)	16 (28.1)	34 (72.3)
Foreigners	14	8.8	3 (42.9)	4 (57.1)	8 (57.1)	5 (62.5)	0 (0)	1 (100)
			Fisher's exact test=0.348, df=1	Fisher's exact test=0.292, df=1	χ^2=0.001, df=1, *p*=0.970	χ^2=0.12, df=1, *p*=0.732	Fisher's exact test=0.724, df=1	Fisher's exact test=0.729, df=1
**Sexual orientation**								
Heterosexual	153	96.2	53 (53.4)	50 (72.5)	84 (57.8)	58 (57.4)	16 (27.6)	35 (72.9)
Homosexual/Bisexual	6	3.8	1 (100)	-	3 (50)	1 (33.3)	-	-
			Fisher's exact test=0.568, df=1	-	Fisher's exact test=0.506, df=1	Fisher's exact test=0.399, df=1	Fisher's exact test=0.724, df=1	-
**Marital status**								
unmarried/widowed/separated/divorced	80	50.3	28 (53.9)	27 (73)	42 (55.3)	28 (54.9)	7 (20.6)	21 (70)
Married/cohabitant	79	49.7	26 (60.5)	23 (72)	45 (60)	31 (58.5)	9 (37.5)	14 (77.8)
			χ^2=0.42, df=1, *p*=0.517	χ^2=0.01, df=1, *p*=0.919	χ^2=0.35, df=1, *p*=0.556	χ^2=0.14, df=1, *p*=0.712	χ^2=2.01, df=1, *p*=0.156	χ^2=0.34, df=1, *p*=0.557
**Sons/daughters**	2.4±1.9 (0-9)*		t-test (93) = 0.482, *p*=0.631	t-test (67) = -0.592, *p*=0.556	t-test (149) = -1.476, *p*=0.142	t-test (102) = -1.918, *p*=0.058	t-test (56) = 0.376, *p*=0.709	t-test (46) = 0.268, *p*=0.790
No	28	17.6	5 (41.7)	5 (55.6)	12 (46.2)	8 (44.4)	1 (12.5)	6 (75)
Yes	131	82.4	49 (59)	45 (75)	75 (60)	51 (59.3)	15 (30)	29 (72.5)
			χ^2=1.29, df=1, *p*=0.256	χ^2=1.48, df=1, *p*=0.223	χ^2=1.69, df=1, *p*=0.194	χ^2=1.34, df=1, *p*=0.247	Fisher's exact test=0.287, df=1	χ^2=0.02, df=1, *p*=0.885
**Education level**								

None/Primary school	43	27	14 (48.3)	18 (72)	18 (43.9)	19 (52.8)	6 (30)	11 (61.1)
Middle school	84	52.8	33 (63.5)	25 (75.8)	48 (60.7)	31 (62)	10 (32.3)	20 (83.3)
High school	28	17.6	5 (41.7)	6 (60)	18 (66.7)	6 (42.9)	0 (0)	3 (60)
University degree	4	2.5	2 (100)	1 (100)	3 (75)	3 (75)	0 (0)	1 (100)
			$\chi^2$for trend =0.49, p=0.485	$\chi^2$for trend =0.05, p=0.831	$\chi^2$for trend =4.34, p=0.037	$\chi^2$for trend =0.06, p=0.813	$\chi^2$for trend =1.4, p=0.237	$\chi^2$for trend =0.83, p=0.363
Detention								
**Institution**								
Prison 1	31	19.5	4 (22.2)	12 (80)	20 (66.7)	11 (57.9)	2 (22.2)	6 (66.7)
Prison 2	128	80.5	50 (64.9)	38 (70.4)	67 (55.4)	48 (56.5)	14 (28.6)	29 (74.4)
			Fisher's exact test=0.001, df=1	χ^2=0.55, df=1, p=0.460	χ^2=1.26, df=1, p=0.262	χ^2=0.01, df=1, p=0.910	Fisher's exact test=0.523, df=1	χ^2=0.22, df=1, p=0.640
**Occupation before detention** [a]								
No	58	37.7	16 (50)	17 (73.9)	34 (62.9)	20 (62.5)	8 (44.3)	14 (93.3)
Yes	100	62.3	38 (60.3)	33 (71.7)	53 (55.2)	39 (54.9)	8 (20)	21 (63.6)
			χ^2=0.92, df=1, p=0.337	χ^2=0.04, df=1, p=0.849	χ^2=0.85, df=1, p=0.356	χ^2=0.52, df=1, p=0.472	χ^2=3.71, df=1, p=0.054	χ^2=4.61, df=1, p=0.032
**First detention** [a]								
No	48	30.4	15 (57.7)	15 (75)	19 (42.2)	14 (42.4)	2 (10)	12 (66.7)
Yes	110	69.6	39 (57.4)	34 (70.8)	68 (64.8)	44 (62.9)	14 (36.8)	23 (76.7)
			χ^2=0.001, df=1, p=0.976	χ^2=0.12, df=1, p=0.727	χ^2=6.57, df=1, p=0.010	χ^2=3.81, df=1, p=0.051	Fisher's exact test=0.027, df=1	χ^2=0.57, df=1, p=0.450
**Length of detention, years** [a]	2.6±4.3 (<1-29)*		t-test (91) =0.651, p=0.516	t-test (65) =-0.938, p=0.352	t-test (147) =-0.773, p=0.441	t-test (100) =1.232, p=0.221	t-test (56) =1.661, p=0.102	t-test (46) =0.125, p=0.901
≤1	75	47.2	23 (52.3)	29 (74.4)	42 (60)	34 (63)	8 (29.6)	17 (68)
2-5	59	31.1	23 (63.9)	13 (65)	31 (54.4)	19 (48.7)	8 (34.8)	12 (75)
>5	25	15.7	7 (53.9)	7 (87.5)	13 (59.1)	5 (55.6)	0 (0)	6 (85.7)
			χ^2=1.15, df=2, p=0.562	χ^2=1.544, df=2, p=0.462	χ^2=0.43, df=2, p=0.808	χ^2=1.88, df=2, p=0.391	χ^2=3.70, df=2, p=0.157	χ^2=0.92, df=2, p=0.631
**Working activity in prison**								
No	99	62.3	33 (55.9)	36 (73.5)	50 (54.4)	41 (57.8)	10 (26.3)	26 (74.3)
Yes	60	37.7	21 (58.3)	14 (70)	37 (62.7)	18 (54.6)	6 (30)	9 (69.2)
			χ^2=0.05, df=1, p=0.819	χ^2=0.09, df=1, p=0.770	χ^2=1.03, df=1, p=0.310	χ^2=0.09, df=1, p=0.759	χ^2=0.09, df=1, p=0.765	χ^2=0.12, df=1, p=0.726
**Type of cell**								
Individual	8	5	1 (25)	2 (100)	6 (75)	1 (50)	0 (0)	0 (0)
Shared	151	95	53 (58.2)	48 (71.6)	81 (56.6)	58 (56.9)	16 (28.1)	35 (74.5)

			Fisher's exact test=0.213, df=1	Fisher's exact test=0.522, df=1	Fisher's exact test=0.261, df=1	Fisher's exact test=0.681, df=1	Fisher's exact test=0.724, df=1	Fisher's exact test=0.271, df=1
Anamnestic								
**At least one chronic disease**								
No	100	62.9	31 (62)	26 (70.3)	57 (58.2)	37 (56.1)	8 (32)	13 (68.4)
Yes	59	37.1	23 (51.1)	24 (75)	30 (56.6)	22 (57.9)	8 (24.2)	22 (75.9)
			χ^2=1.14, df=1, *p*=0.285	χ^2=0.19, df=1, *p*=0.661	χ^2=0.03, df=1, *p*=0.853	χ^2=0.03, df=1, *p*=0.856	χ^2=0.43, df=1, *p*=0.513	χ^2=0.32, df=1, *p*=0.571
**Cardiovascular disease**								
No	127	79.9	40 (59.7)	37 (74)	72 (59)	43 (52.4)	12 (30.8)	20 (64.5)
Yes	32	20.1	14 (50)	13 (68.4)	15 (51.7)	16 (72.7)	4 (21.1)	15 (88.2)
			χ^2=0.76, df=1, *p*=0.384	χ^2=0.22, df=1, *p*=0.643	χ^2=0.51, df=1, *p*=0.475	χ^2=2.91, df=1, *p*=0.088	Fisher's exact test=0.327, df=1	χ^2=3.13, df=1, *p*=0.077
**Diabetes**								
No	148	93.1	48 (56.5)	42 (71.2)	85 (59)	56 (57.7)	12 (24.5)	29 (72.5)
Yes	11	6.9	6 (60)	8 (80)	2 (28.6)	3 (42.9)	4 (44.4)	6 (75)
			χ^2=0.05, df=1, *p*=0.831	χ^2=0.33, df=1, *p*=0.564	Fisher's exact test=0.116, df=1	Fisher's exact test=0.351, df=1	Fisher's exact test=0.201, df=1	χ^2=0.02, df=1, *p*=0.885
**Body Mass Index (BMI) category** [a]	27.8±6.8 (14.7-49.8)*		t-test (90) =-1.821, *p*=0.072	t-test (65) = -0.896, *p*=0.374	t-test (141) = -0.674, *p*=0.501	t-test (97) = -0.144, *p*=0.886	t-test (55) = -2.059, *p*=0.044	t-test (45) = -0.578, *p*=0.566
Underweight/Healthy weight	64	42.4	20 (57.1)	19 (76)	31 (50)	27 (64.3)	5 (21.7)	14 (70)
Overweight	37	24.5	14 (53.9)	13 (61.9)	26 (76.5)	13 (54.2)	5 (27.8)	10 (76.9)
Obese	50	33.1	20 (64.5)	16 (76.2)	27 (57.5)	17 (51.5)	6 (37.5)	10 (71.4)
			χ^2=0.72, df=2, *p*=0.698	χ^2=1.43, df=2, *p*=0.490	χ^2=6.40, df=2, *p*=0.041	χ^2=1.38, df=2, *p*=0.500	χ^2=1.16, df=2, *p*=0.559	χ^2=0.2, df=2, *p*=0.906
Lifestyle								
**Smoking status**								
Never smoker	35	22	14 (66.7)	12 (75)	20 (60.6)	15 (62.5)	5 (45.5)	10 (100)
Former smoker	13	8.2	3 (33.3)	3 (50)	6 (50)	5 (55.6)	2 (40)	3 (75)
Current smoker	111	69.8	37 (56.9)	35 (74.5)	61 (57.5)	39 (54.9)	9 (21.4)	22 (64.7)
			χ^2=2.85, df=2, *p*=0.240	χ^2=1.66, df=2, *p*=0.435	χ^2=0.41, df=2, *p*=0.816	χ^2=0.42, df=2, *p*=0.809	χ^2=2.94, df=2, *p*=0.230	χ^2=4.88, df=2, *p*=0.087
**Number of cigarettes/ day**[b]	20.1±10.2 (2-60)*		t-test (63) =0.555, *p*=0.581	t-test (45) =0.233, *p*=0.817	t-test (104) =0.251, *p*=0.802	t-test (69) =0.062, *p*=0.951	t-test (40) =0.738, *p*=0.464	t-test (32) =0.189, *p*=0.851
**Alcohol consumption** [a] **(AUDIT-C)**								
Never	83	53.2	33 (57.9)	29 (70.7)	47 (60.3)	32 (56.1)	10 (26.3)	26 (78.8)

Not being at risk of alcohol abuse	45	28.9	16 (61.5)	16 (76.2)	24 (55.8)	17 (54.8)	4 (26.7)	7 (58.3)
Being at risk of alcohol abuse	28	17.9	5 (55.7)	3 (60)	16 (57.1)	8 (57.1)	1 (33.3)	1 (50)
			χ^2=0.14, df=2, p=0.933	χ^2=0.56, df=2, p=0.754	χ^2=0.25, df=2, p=0.884	χ^2=0.02, df=2, p=0.988	χ^2=0.07, df=2, p=0.966	χ^2=2.36, df=2, p=0.307
**Physical activity status [a] (IPAQ)**								
Inactive	47	29.9	17 (56.7)	17 (80.9)	25 (59.5)	16 (61.5)	6 (34.6)	11 (78.6)
Minimally active	67	42.7	21 (53.8)	14 (50)	35 (53)	22 (47.8)	1 (4.5)	15 (71.4)
Active/very active	43	27.4	16 (66.7)	19 (100)	27 (64.3)	21 (67.7)	8 (53.3)	9 (75)
			$\chi^2$for trend =0.47, p=0.494	$\chi^2$for trend =1.53, p=0.217	$\chi^2$for trend =0.19, p=0.659	$\chi^2$for trend =0.33, p=0.568	$\chi^2$for trend =1.40, p=0.237	$\chi^2$for trend =0.05, p=0.820
**At least 5 daily portions of fruit and vegetables**								
No	49	30.8	12 (54.5)	10 (66.7)	21 (46.7)	12 (38.7)	1 (7.1)	9 (75)
Yes	110	69.2	42 (57.5)	40 (74.1)	66 (62.3)	47 (64.4)	15 (34.1)	26 (72.2)
			χ^2=0.01, df=1, p=0.912	χ^2=0.32, df=1, p=0.570	χ^2=3.15, df=1, p=0.076	χ^2=5.84, df=1, p=0.016	Fisher's exact test=0.045, df=1	χ^2=0.04, df=1, p=0.851
**Two daily portions of dietary protein sources**								
No	93	58.5	31 (56.4)	27 (71.1)	52 (57.8)	32 (52.5)	6 (21.4)	14 (66.7)
Yes	66	41.5	23 (57.5)	23 (74.2)	35 (57.4)	27 (62.8)	10 (33.3)	21 (77.8)
			χ^2=0.01, df=1, p=0.912	χ^2=0.08, df=1, p=0.771	χ^2=0.01, df=1, p=0.961	χ^2=1.09, df=1, p=0.295	χ^2=1.03, df=1, p=0.311	χ^2=0.74, df=1, p=0.390
**Rare snacks and sweets consumption**								
No	76	47.8	22 (56.4)	20 (80)	45 (60.8)	29 (56.9)	5 (23.8)	14 (82.4)
Yes	83	52.2	32 (57.1)	30 (68.2)	42 (54.5)	30 (56.6)	11 (29.7)	21 (67.7)
			χ^2=0.01, df=1, p=0.943	χ^2=1.12, df=1, p=0.291	χ^2=0.61, df=1, p=0.436	χ^2=0.0007, df=1, p=0.979	χ^2=0.24, df=1, p=0.628	χ^2=1.19, df=1, p=0.276

*Mean±Standard Deviation (Range)

[a] Number of each item may not add up to total number of study population due to missing values.

[b] In current smokers.

[c] Papanicolaou test.

[d] Fecal occult blood test.

[#] Age 45-69 year.

▲Only among those who have not yet undergone it in prison.

• Age 25-64 year.

◊ Age 50-69 year.
